# Older Adults’ Demand for Integrated Care and Its Influencing Factors: A Scoping Review

**DOI:** 10.5334/ijic.5946

**Published:** 2021-12-06

**Authors:** Zhenyu Wang, Zhihan Liu

**Affiliations:** 1 School of Public Administration, Central South University, Changsha, Hunan Province, China

**Keywords:** integrated care, needs, influencing factors, scoping review, older adults

## Abstract

**Introduction:**

Integration has become a major concern for governments, healthcare and aged care systems in many countries. However, the research on and implementation of integrated care in China started relatively late, and there is no review on the needs of older adults with regard to integrated care and the influencing factors. Therefore, this paper aims to provide a scoping review by searching, evaluating, and summarizing the Chinese and international literature on the need for and the factors influencing integrated care for older people. In addition, this review highlights evidence of the gap between China and the world in integrated care.

**Methods:**

Using a framework proposed by Arksey and O’Malley, a systematic search of 12 domestic and international databases was conducted. Of the 890 original studies retrieved, those that met the established inclusion criteria were screened and scored using the Ekman quality assessment tool. The qualitative description method was used to summarize the demand for integrated care for older adults and the influencing factors.

**Results:**

A total of 49 papers were included. These studies were from eleven countries on five continents (most commonly China and the US) and were mostly cross-sectional quantitative studies that surveyed the integrated care needs of older people living in homes/communities or long-term care facilities. The analysis shows that existing research on the integrated care needs of older people in China adopts a single perspective and is inadequate and unsystematic in its assessment; the integrated care needs of older adults and the factors influencing them are multifaceted; and both in China and internationally, the community-home care scenario most consistently meets the needs and expectations of older adults.

**Conclusion:**

Although there is no uniform definition of integrated care in China or abroad and each country has its own national definition and system of integrated care, there are certain commonalities regarding the needs of older adults and the factors that influence them across countries. Our research reveals a gap between China and the international community in terms of integrated care.

## Introduction

In recent years, the ageing trend has become prominent in China. Compared with European developed countries and the United States, China shows a unique ageing pattern of “Ageing before Affluence”, i.e., the rate of population ageing exceeds the rate of per capita income increase [1]. Additionally, ageing in China is occurring on a larger scale and at a more rapid pace, creating a heavier dependency burden. The traditional nursing model cannot comprehensively satisfy the complicated care needs of older Chinese adults [2]. As family support functions continue to weaken and older people’s demand for professional nursing and health services continues to increase, opportunities for innovative older adult care models are emerging [3]. Integrated care has become an ideal model to meet the increasingly complex care needs of older adults by virtue of its high quality, stability, and economy [4].

In China, ‘*Yiyang Jiehe*’—which means “health and social care combination” or “medical and older adults care combination”—has become synonymous with integrated care for older people [2,5]. The Chinese government has issued a series of relative policies [5,6] and designated 90 national pilot cities and more than 300 provincial pilot cities to promote the development of this emerging care model. An increasing number of service delivery modes have been proposed, tested and proven to be effective during this process, e.g., collaboration between medical and older adult care providers or expansion of the service scope of either medical or older adult care providers to cover all services by increasing facilities and personnel [2].

Due to their rapid economic development and the early emergence of ageing problems, some developed countries established an institutionalized, large-scale, and increasingly mature integrated care model for older adults as early as the last century through pilots, practice and extension [7,8]. The most typical model of community integrated care in the United States is the Program of All-Inclusive Care for the Elderly (PACE) [9]. In addition, there are community-based supports and services (CBSS) [10] and community-based adult services (CBAS) [11]. Since the 1970s, in the United Kingdom, the “integration of health and social care” has been an important policy goal of the government. Through this effort, community-level service areas, service content, service organizations, and professionals have been integrated [12]. The Japanese integrated care model is characterized mainly by the long-term care insurance (LTCI) programme and a sound legal system [13]. In the Netherlands, Embrace is an integrated care service designed for all community-living older adults and combines the Chronic Care Model (CCM) with risk profiles based on a population health management model [14]. The aim is to improve the health outcomes of older adults and to modify the factors that may influence these health outcomes [15]. In addition, Singapore set up the Agency for Integrated Care (AIC) to provide integrated care and long-term care (LTC) services for older adults and their caregivers in 2009 [16]. Due to differences in national conditions, medical and health care and older adult care service systems across countries, the definition of integrated services also differs, and a wide range of definitions and concepts of integrated care exist [17]. Regardless of the country, the core aim of these services is to provide integrated services that allow older persons to enjoy their later life, so these services are collectively referred to as “integrated care” in this article.

To achieve the Global Strategy and Plan of Action for Active and Healthy Ageing, the World Health Organization (WHO) published the Integrated Caring for Older People (ICOPE) guidelines in 2017, which aim to provide community, primary and secondary health care providers with evidence-based recommendations for preventing, delaying or reversing physical and mental decline in older people [18]. The WHO defines integrated care as ‘services that are managed and delivered so that people receive a continuum of health promotion, disease prevention, diagnosis, treatment, disease management, rehabilitation and palliative care services, coordinated across the different levels and sites of care within and beyond the health sector, and according to their needs throughout the life course’ [19]. In contrast, China’s ‘*Yiyang Jiehe*’, which is focused on exploring models of integrated care and their pathways to realization and involves four levels: nursing home, home, community and hospital [2,5]. The WHO-ICOPE is based mainly on the community level and aims to provide more detailed guidance for the development of integrated care in each country. Although both the WHO-ICOPE and China’s ‘*medical-nursing combination*’ are people-centred [20], the WHO-ICOPE is more explicit regarding the application of the framework on integrated people-centred health services in the context of care for older people [21]. Additionally, the WHO-ICOPE is based on the needs of older people, and therefore, the ICOPE guidelines state that services must be oriented towards the needs of older people, whether they have a high and stable level of intrinsic capacity, are declining in function, or are deteriorating in function and require care and support [18,22].

As the WHO-ICOPE has noted, the needs of older adults should be fully understood before providing integrated care services. However, as older people age, their health problems tend to become more chronic and complex, with multimorbidity, the coexistence of multiple chronic conditions, becoming the norm rather than the exception [22]. Older adults are also more likely to suffer from depressive symptoms (DS) [23]. In addition to medical and health needs, older people often have nutritional needs, safety needs and social participation needs [18,24]. The complex physical and mental health conditions that older people present with can result in multifaceted, integrated needs that are difficult to identify and meet. Identifying older people with unmet health needs is a key challenge for aged care providers worldwide [25]. Several studies have suggested that unmet needs precede functional decline, undermine older adults’ ability to manage their daily functioning and increase the risk of emergency room visits, hospital admissions, nursing home placement and premature mortality [26,27,28]. Older people are more than the vessels of their disorders or health conditions; they are individuals with unique experiences, needs and preferences [19]. Various studies have proven the importance of assessing the integrated care needs of older adults.

In recent years, research on integrated care in China has focused on the connotations of the model, the existing problems and the realization path, while less attention has been given to the cognition and demand of the older population with respect to integrated care [2,29]. Older people’s health and social care needs are not well recognized, understood or met. As service targets, the integrated care needs of older adults play a decisive role in the development of integrated care services [30,31]. Furthermore, little is known regarding the factors that influence care recipients’ and families’ decisions to utilize integrated care services [17]. To our knowledge, there is no review on older people’s need for integrated care and the factors that influence it. Moreover, the research and implementation of integrated care in China has lagged that of other countries, so integrated care is still an imperfect and unsystematic novelty in China. Therefore, a comprehensive review is require to identify the gap between China and the remainder of the world through the evaluation, comparison and summary of domestic and international literature to provide information and evidence that will allow China to formulate measures to meet the integrated care needs of its senior citizens and thereby improve their quality of life.

Because the scoping review method is flexible and allows qualitative and quantitative research to be included, a variety of topics can be explored without restriction, such as the needs of patients and caregivers and the determinants of health. It can also be used to identify existing gaps in the literature. These gaps [32,33] are in line with the premise of this study that there is no uniform conclusion regarding “integrated care”, either domestically or internationally. Based on this premise, we conducted a scoping review by searching domestic and foreign documents on the integrated care needs of older adults and the factors influencing them and used the scoping review method to analyse and sort the data.

## Methods

This study adopts the framework proposed by Arksey and O’Malley [32] and refined by Levac et al. [34]. There are 6 steps in total. Below, we briefly summarize each step.

### Step 1: Identify review question

The purpose of this review was noted above. At present, there is no uniform conclusion regarding ‘integrated care’, either domestically or internationally. For the purposes of this review, we have combined the WHO descriptions [19] with Leutz’s [35] perspectives to define integrated care as

“a process to overcome fragmentation, based on a person-centred approach that connects the health care system (acute, primary care and skilled) with other human service systems (such as long-term care, education and vocational and housing services) to improve outcomes (clinical, satisfaction and efficiency) through different levels and sites of care and service delivery to meet people’s needs”.

The review attempts to answer three research questions:

1. In integrated care, what are the specific service needs of older adults?
2. What factors (such as gender, age, health status, or number of children) affect older adults’ needs of for integrated care services?
3. In research and services on integrated care, what are the differences in the needs of older people in China and abroad and their influencing factors? What gaps exist?

### Step 2: Identifying relevant literature

Published and unpublished (‘grey’) literature were identified based on a comprehensive three-step search strategy recommended by the Joanna Briggs Institute (JBI) systematic reviews [36]. In the first step, through the Central South University (CSU) online library, we conducted an initial limited search of the foreign language database Web of Science and the Chinese database China National Knowledge Infrastructure (CNKI) and then analysed the text words contained in the titles and abstracts as well as the English and Chinese index terms used to describe the articles. A second search was conducted in all included databases using all identified keywords and index terms. Additional studies were searched in the reference lists of all identified articles.

Chinese search strategy: Five Chinese databases were used, namely, the China National Knowledge Infrastructure (CNKI), Wang Fang Data, VIP Chinese Database (VIP), Chinese Scientific Document Service System (CSCD) and Chinese Social Science Citation Index (CSSCI), were searched. Foreign language search strategy: Seven English databases were used, namely, Web of Science, Elsevier/Science Direct, MEDLINE, PubMed, Springer Link, EBSCO ASP, and The Cochrane Library. A manual search was also performed with Baidu Academic and Google search. We did not limit publication dates or language in the searches. We consulted a scientific librarian for the synonyms and search strategies for each database (see ***Table 1*** for example search strategies from one database).

**Table 1:** Specific search terms and search strategies.


KEY SEARCH TERMS		ALTERNATIVE SEARCH TERMS

Integrated care	OR	“combination of medical care and pension*”.ti, ab., “medical-nursing combination*”.ti, ab., “medical and older adults care combination*”.ti, ab., “PACE*”.ti, ab., “NHS*”.ti, ab., “long-term care”.ti, ab., “long-term nursing”.ti, ab., “integrated long-term care”.ti, ab., “Health Service”.ti, ab., “home-and community-based services (HCBS)*”.ti, ab., “community-based adult services(CBAS)*”

AND needs	OR	“demands”.ti, ab., “choice”.ti, ab., “preference”.ti, ab.

AND older adults	OR	“older adult*”.ti, ab., “older people”.ti, ab, elderly.ti, ab., “ageing population*”.ti, ab., senior.ti, ab.

AND influencing factors	OR	“affect”.ti, ab., “influence”.ti, ab.


### Step 3: Study selection

**Figure 1:**
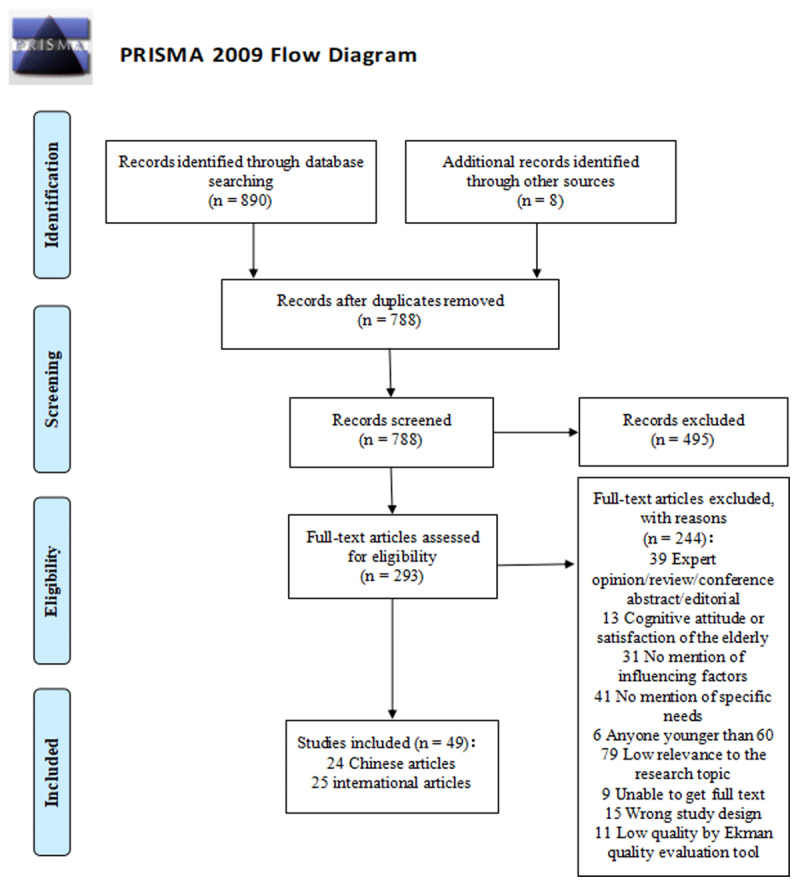
Literature inclusion process and results.

Studies were selected through a two-step process using the selection criteria below. Two members of the research team independently reviewed the titles and abstracts of each study according to the literature inclusion criteria to complete the preliminary screening. The two team members then carefully read the full text of the articles and used the exclusion criteria to evaluate and complete the secondary screening. If there were differences of opinion, another researcher on the team was consulted to obtain a consistent final result. In addition, during this review, guided by the PRIMSA statement for systematic review protocols [36], a flowchart was created (see ***Figure 1***).

The inclusion criteria were as follows: (1) the needs of older adults for integrated care and the influencing factors were studied, and the relevant research results were summarized; (2) the study involved older adults aged ≥60 years; (3) the service location included in the literature could be any setting, including a medical or health institution, an older adult care institution, a community, or a home; and (4) any type of primary study (quantitative, qualitative or mixed methods) was performed.

The exclusion criteria were (1) reviews, press releases, commentaries, conference briefs, and science fiction; (2) publications concerning older people’s perceived attitudes (acceptance or lack thereof rather than need) or satisfaction with integrated care; (3) duplicates or literature for which full-text information was unavailable; (4) literature that was less relevant to the content of the study; (5) literature that lacked either of the two themes of demand for or factors influencing integrated care; and (6) literature that was rated as low quality by the Ekman quality assessment tool [37].

Although the scoping review did not have strict requirements for quality control of the literature, this study used the Ekman quality assessment tool to evaluate the quality of the included literature. The tool was initially used to evaluate studies on community health services insurance in low- and middle-income countries [37] and covers seven dimensions: analytical question(s), rationale, methodology, data, goal achievement, findings and results, and discussion and conclusions. A score of 0 on this quality rating scale means that the article did not implement the criterion at all, a score of 1 means that the article partially implemented the criterion, and a score of 2 means that the article fully implemented the criterion. Only articles that used statistical regression analysis received a score of 3 on the third question about methodology [38]. An overall score of 22–25 (3 stars) indicates high quality, a score of 17–21 (2 stars) indicates moderate quality, and a score of 0–16 (1 star) indicates low quality. The quality of each included document was evaluated by two independent researchers back-to-back. In the case of disagreement, another researcher on the team was consulted until agreement was reached.

### Step 4: Charting the data

Microsoft Excel software was used to extract data, and bibliometric methods were applied. The final information included the following three categories: (1) literature information: country, first author, publication year, journal name, journal level, article type, and research method; (2) key content: service venue, integrated care needs and influencing factors; and (3) survey information: survey population and sample size, recruitment and data collection methods, inclusion and exclusion criteria, survey duration, and related information. Charting should be an iterative process in which researchers continually extract data and update the data-charting form [34].

### Step 5: Collating, summarizing, and reporting the results

Based on the description of Arksey and O’Malley [32] and Levac et al. [34], this step should include three substeps. First, based on the existing framework, a descriptive numerical summary and analysis of the included literature is performed to describe the characteristics of the included research (e.g., type of research design, year of publication, type of intervention, characteristics of the research population, the country where the research is conducted). Second, the results of the scoping study are described with themes, a framework, or a table identifying strengths and gaps in the evidence. Finally, the significance of the results and the broader implications for research, policy, and practice are considered.

In this scoping review, we sought information from older adults and their caregivers about integrated care needs and the factors influencing them in older adults. In terms of integrated care needs, we referred to the service content and framework in the “Guidelines for the Management of Integrated Medical Care and Care Institutions (Trial)” [39] issued by China in 2019 to clarify integrated care needs. We then classified and summarized the actual integrated care needs included in the literature. In terms of factors influencing the need for comprehensive care, this study modifies and extends the WHO framework of determinants of active ageing [24] and differentiates the determinants according to the classification of the included literature. In summary, this review used a qualitative descriptive approach to summarize the needs of older people for integrated care and the factors that influence them.

### Step 6: Consultation

Consultation and member checking are optional when conducting scoping reviews [33]. However, this research performs this step. It considers consultation with stakeholders as the necessary research content and collects relevant information to ensure the quality of the research results. We invited older people (n = 3, two from Meixi Lake Jinmao Community and one from Wanfo Hill Nursing Home), persons in charge of community-aged home service centres (n = 2, both from Meixi Lake Jinmao Community), persons in charge of nursing homes (n = 2, both from Wanfo Hill Nursing Home), and formal caregivers (n = 2, both from Wanfo Hill Nursing Home) to participate in stakeholder consultation meetings in the form of semi-structured interviews on 10 January 2021 in Changsha. As both Meixi Lake Jinmao Community Aged Home Service Centre and Wanfo Hill Nursing Home are the research partners of our research team, we communicated with the heads of these two institutions so that we could invite all stakeholders to a stakeholder consultation meeting at Wanfo Hill Nursing Home. We clarified the goal of jointly discussing the integrated care needs of older adults and their influencing factors, shared the preliminary results from the literature review stage, and asked whether there were any integrated care needs and influencing factors not emphasized in our results to improve our research framework. All team members participated in Stage 6 of the review to ensure an appropriate fit between the questions posed and sources of evidence gathered, thus confirming that the method was clearly justified and the analysis was accurate.

## Results

By applying the search strategy, out of 898 citations retrieved, 110 were duplicates, leaving 788 for title/abstract and full text screening, resulting in 49 articles (24 articles in Chinese and 25 articles in English) included in this review. Of these, 25 are high quality (3 stars) [2,25,40,41,42,43,44,45,46,47,48,49,50,51,52,53,54,55,56,57,58,59,60,61,62], and 24 are medium quality (2 stars) [5,63,64,65,66,67,68,69,70,71,72,73,74,75,76,77,78,79,80,81,82,83,84,85]. The perspectives of older adults and their caregivers were considered when identifying the needs of older adults with regard to integrated care. The following sections highlight the characteristics of the studies, the details of the study participants across all the studies reviewed and the thematic analysis of the findings. Key information from each study is shown in Appendix 1.

### Characteristics of the included studies

The earliest journal article was published by Li [54] in 2004, and the included papers were published between 2004 and 2020. The study areas covered include 11 countries on five continents. Of the studies conducted in Asia, twenty-eight studies were conducted in China [2,5,46,47,49,55,58,60,61,62,65,66,67,69,71,72,73,74,75,76,77,78,79,81,82,83,84,85], one in Lebanon [63] and one in Japan [50]. One was conducted in Canada [64], and nine were conducted in the US [40,41,42,44,45,52,53,54,70]. Of the studies conducted in Europe, three were performed in Germany [43,57,80], two in the Netherlands [48,59], one in Ireland [51], and one in Sweden [56]. Finally, 2 studies were conducted in New Zealand/Australia [25,68]. Six are master’s degree theses [47,49,55,62,67,77], and the remainder are journal articles. In terms of integrated care needs assessment tools for older people, 8 articles used only 1 type of well-established scale to conduct surveys [51,53,62,64,71,73,75,78], 12 papers used 2–4 types [25,40,41,42,45,48,50,55,57,63,74,80] and 3 papers used 5–7 types [44,59,68]. Only 1 article did not use any scales [54]. However, we found that only 5 foreign scholars used self-compiled questionnaires [43,52,53,56,70]. The “well-established scale” was meant to refer to scales that are not self-compiled, widely used, validated and stable. In contrast, more than half of Chinese scholars used self-compiled questionnaires that were unvalidated or not widely used [2,5,46,47,49,55,58,60,61,62,65,66,67,69,71,72,73,76,77,78,79,81,82,83,84,85]. In addition, there were only 2 mixed method studies [52,62], and the remainder were quantitative studies (including 45 cross-sectional and 2 longitudinal studies).

We also found that the sample size of the included studies ranged from a minimum of 56 people [51] to a maximum of 7,320 people [2]. Two studies focused only on informal caregivers, such as spouses and children of older adults [42,54], 5 studies interviewed older adults and their caregivers [41,43,59,68,80]; two of these five studies investigated formal caregivers, such as qualified nurses and nursing staff [59,80]. However, the survey subjects in the Chinese studies were all older adults; caregivers were not interviewed. Of the included literature, most studies (n = 29) were conducted in the service scenario of “Home and Community” [25,40,41,42,43,44,45,46,48,50,52,53,54,55,56,62,63,64,66,68,70,73,74,75,77,78,82,83] (with 1 community-based LTC facility [71]), and 11 settings were integrated care or long-term care institutions [47,51,57,58,59,60,72,76,80,81,84]. Studies that did not define or distinguish service venues were performed by Chinese scholars [2,5,49,61,65,67,69,79,85].

### The need for integrated care among older adults

After classifying and summarizing the actual integrated care needs of older people in the included literature, 8 categories of needs that make up the integrated care needs of older people emerged: basic life, medical and nursing, rehabilitation, ancillary, psycho-spiritual support, social participation, health education, and welfare and aid.

For basic life needs, there are 13 subcategories under this category, mapping 43 articles that cover nearly all the basic needs of older people in their daily lives. Of these needs, self-care, housekeeping and meal preparation were the most commonly identified service needs, covering 31, 25 and 19 articles in that order. Six articles referred to the sensory needs of older people in terms of sight, hearing and communication, while sensory needs and self-mobility were discussed simultaneously in 4 articles. However, both sleep aids and memory were only mentioned in 2 articles. The need for access to medical and nursing needs was a category that emerged most often in the studies reviewed; 17 subcategories and 45 articles were included. Of these, 21 articles explored the home health care needs of older people. Disease diagnosis and treatment (n = 18), regular physical examination (n = 14), skilled nursing (n = 11) and medication (n = 11) are all high-frequency needs. However, both emergency care and skin care were discussed in only 1 article each. In total, 14 articles explored the rehabilitation needs of older people, with 10 additional articles addressing rehabilitation and health care rather than rehabilitation guidance. Within the category of ancillary needs, six sub-categories and 19 articles were found, and a relatively high number of articles explored the four subcategories of transportation (n = 8), shopping (n = 5), older adults care hotline (n = 5), and managing money (n = 5); Among psycho-spiritual support needs, there were a large number of articles on spiritual solace, psychological counselling, and company, representing 12, 15, and 12 articles, respectively. The social participation needs category includes 10 subcategories, with cultural and entertainment service (CES) being the most discussed subcategory with 17 articles included. Among health education needs, both health guidance and health lectures appeared in 5 articles, while the three needs of health guidance, health lectures and health training were mentioned simultaneously in Liu’s [55] dissertation. Finally, 9 articles were mapped to the welfare and aid needs category, while the number of articles exploring legal aid was two more than that of social welfare (see ***Table 2*** for a description of each subcategory).

**Table 2:** A summary of various categories of integrated care needs among older adults.


CATEGORY	SUBCATEGORY AND STUDIES	DESCRIPTION

Basic life needs	Accommodation [25,48,54,58,59,63,70,78,81]	Inappropriately or inadequately housed, e.g., adaptation required, home repair, vacuuming; gardening; maintenance; lawn mowing; add decorations in the living space

	Preparing meals [5,25,43,44,45,48,49,54,55,56,59,62,63,64,68,70,78,79,84]	Prepare breakfast and cooking meals and provide home-delivered and congregate meals; ensure the good nutritional status of older adults

	Housekeeping services [5,25,41,42,44,48,49,50,52,54,56,58,59,60,62,68,70,74,75,78,79,81,82,83,84]	Assistance with chores/homemaking, cleaning, tidying up, completing the laundry and errands

	Self-care [2,5,25,42,43,47,48,49,50,53,55,56,58,59,60,61,63,64,66,67,68,69,71,74,75,76,78,79,81,82,84]	Management of hygiene, including eating, bathing, shampooing, shaving, nail trimming, dressing, grooming, using the toilet

	Sensory needs [48,57,59,63,64,80]	Eyesight/hearing/communication/watching TV

	Daytime activities [57,59,63,80]	Helping older adults participate in regular appropriate daytime activities

	Self-mobility [48,56,59,63,64,68]	Transfers, walking inside, walking outside, using the stairs, putting on prostheses or orthoses, moving around in a wheelchair

	Adult day care [40,41,42,43,50,54,78]	A health care service provided for adults who require partial or supplemental care and companionship during the day when family members are working or otherwise unable to stay at home with an older adult relative. Among the services that may be offered at an adult day care centre are nursing services (e.g., medication administration and health monitoring); nutritional and health education, health counselling; physical, speech, and occupational therapy; and socialization.

	Respite care [41,42,43,50,54]	Respite care provides short-term relief for primary caregivers. It can be arranged for an afternoon or for several days or weeks. Care can be provided at home, in a healthcare facility, or at an adult day care centre.

	Safety [40,56,72,81]	Emergency response systems and client protection (day and evening) to ensure that everything is OK and that no accidents occurred

	Sleep aids [49,81]	Guidance to help older adults improve their sleep in a variety of ways

	Memory [48,63]	Helping older adults remember recent events and where they placed items

	Caring for others [48,59,63]	Replace older adults or help them care for the people they have responsibility for

Medical and nursing needs	Medical transportation [52,55]	In emergencies, older adults can use professional medical transportation services and receive prioritized medical treatment, or they can be transferred from the community to a higher-level hospital for treatment

	Home health care [2,5,40,41,42,43,44,46,50,55,60,62,68,70,72,73,74,76,78,79,82]	A commonly used bridge strategy for transitioning from hospital to home-based care is expected to contribute to readmission avoidance efforts. Older people can receive home visits from doctors, nurses and physiotherapists or occupational therapists from regional health care facilities.

	Medication [45,48,49,52,55,56,59,63,64,72,81]	Medication reminders and supervision

	Building health archives [46,49]	After conducting a health check-up, record older patients’ physical symptoms and past health (disease, treatment, medication) and establish and maintain health archives.

	Preventive care [60,65,84]	Help the older adults avoid illness through prevention.

	Regular physical examination [5,46,49,55,60,61,66,70,75,78,79,81,82,84]	Regular (once or twice a year) physical examination of older adults through medical means and methods

	Disease diagnosis and treatment [2,5,48,50,55,58,60,63,65,66,67,69,71,73,76,81,84,85]	A doctor conducts professional diagnosis and treatment of older adults.

	Emergency care [85]	Provide emergency response for unexpected serious health events and unexpected security incidents, such as offering assistance to older patients who experience sudden cardiovascular and cerebrovascular events

	Home sickbed care [47,55,61]	According to the treatment needs of older patients and the lifestyle of bedridden persons, the home is used as the nursing site, and medical treatment or rehabilitation is provided in the family environment for certain diseases so that patients can receive medical treatment and nursing in a familiar environment.

	Traditional Chinese medicine services [55,73]	A series of services such as prescribing and brewing Chinese medicine for older adults and providing Chinese medicine treatment

	Palliative care [49,76,78,79]	To assist dying patients and their families while reducing the physical pain of the patient, attention is given to the patient’s inner feelings so that he or she can complete the journey of life with dignity

	Skilled nursing [49,55,59,66,67,71,75,77,78,83,85]	Provided by skilled nurses, this service includes measurement of four vital signs (body temperature, pulse, respiration, blood pressure), injections, dressing changes, and related services.

	Adaptive equipment [40,50,52,54]	All non-insured durable medical equipment, including hearing and visual aids, incontinence supplies, diabetic supplies, and home occupational therapy equipment exclusive of equipment in the home safety category.

	Medical information [40,48,55,59,63,80]	Verbal or written information on one’s condition, medication or treatment

	Chronic disease management [55]	For older adults with chronic diseases, services such as chronic disease screening, follow-up, and guidance on chronic disease self-management are provided

	Accompany to doctor’s visits [55,62,79]	Community staff accompany unaccompanied older patients to doctor visits.

	Skin care [78]	Activities and interventions designed to maintain the integrity of the integument, including care for pressure ulcers and massage.

Rehabilitation needs	Rehabilitation and health care [50,55,62,66,68,71,74,78,79,81,82,84]	A comprehensive service integrating sports therapy, occupational therapy, speech therapy, physical therapy, acupuncture, cupping, and massage

	Rehabilitation guidance [47,55,77,78]	Guidance for older adults and their caregivers on rehabilitation and health care

Ancillary needs	Transportation [25,40,42,44,45,52,54,70]	Public transportation services, such as taxi vouchers, curb-to-curb transportation (i.e., Dial-a-Ride), and volunteer transport, or help applying for a disability placard

	Shopping [25,56,61,62,79]	Shopping for groceries and personal items

	Older adults care hotline [45,46,61,76,78]	Provide support, information, advice, or referrals for older adult callers through a telephone hotline

	Managing money [25,52,59,63,70]	Budget management, banking, paying bills

	Counselling [40,43,55]	Answer questions for older adults

	On-site service [43,55,70]	When older clients require a service at home, community workers are on call to meet their needs

Psycho-spiritual support needs	Spiritual solace [51,52,58,63,65,66,68,69,74,77,78,84]	Provide relief for mental disorders and alleviating mental stress for older adults to meet their daily mental health needs (providers are volunteers)

	Psychological counselling [2,47,49,51,55,57,59,60,62,63,67,78,79,80,81]	Provide mental health and adjustment services to older patients and consulting with them (providers are professional counsellors)

	Company [48,52,59,60,61,62,63,72,77,79,80,84]	Provide emotional support and communication and relieving emotional loneliness by chatting with older adults face-to-face

	Home-like atmosphere [51,81]	Help older people feel like they are living at home using various methods

	Family visits [81,84]	Adult children regularly visit the older adults in nursing homes or at home

Social participation needs	Social support [56,58,68]	Maintain friendship/socialization, help older adults feel cared for and supported by going on walks or talking, having regular telephone interviews, and performing related activities

	Intimate relationships [57,59,63,80]	There is one person or several people on whom the older adults depends, whom they trust, and whom they are willing to tell the truth

	Cultural and entertainment service (CES) [2,5,44,49,59,60,62,63,66,71,74,75,76,77,78,79,81]	Chess and mahjong, drama, singing and dancing, calligraphy and painting, daily reading, etc.

	Sporting fitness [46,62,68,76,78]	Provide older adults individuals with places and opportunities to exercise

	Volunteer activity [49,77]	Organize older citizens to serve in society as much as they can voluntarily and without asking for a reward

	Senior University/Centre [49,62,70,77,78]	Through rationalized course arrangement, provide older adults with places to conduct learning activities in the community

	Support group [41,43]	Mutual aid groups or mutual aid support groups composed of older adults who have the same difficulties

	Employment [70,77]	Introduce older workers to suitable jobs and help them realize their self-worth

	Remarriage [62]	Help divorced, widowed, or unmarried older people connect with new partners and remarry

	Staff make extra effort [51]	Mainly refers to older people’s desire that the staff in the nursing facility will redouble their efforts

Health education needs	Health guidance [5,43,55,60,61,66,67,77,78,82]	Provide education on lifestyle, nutrition and disease management

	Health lectures [5,46,49,55,62,77,78,82,84]	Conduct lectures on health or other useful knowledge for the older population

	Health training [49,55,68]	Provide health knowledge training opportunities for older adults, such as first aid knowledge or life skills training

Welfare and aid needs	Social welfare [62,63,75,77,81]	Medicaid enrolment, endowment insurance, various welfare allowances, and government subsidies

	Legal aid [40,44,61,62,70,75,77]	Provide legal services on civil matters to older adults, e.g., assistance with wills and end-of-life documents


### Factors influencing the integrated care needs of older adults

After collating and summarizing the influencing factors obtained through various analytical methods in the included literature, it was found that the factors affecting the integrated care needs of older adults can be divided into six categories: demographic, personal, psychological, family, community, and social. Of these, 33 articles explored demographic factors, and the age (n = 19), gender (n = 11), educational attainment (n = 15), preretirement occupation (n = 7) and marital status (n = 9) of older adults were the main demographic factors that influenced integrated care needs. Among the personal factors, the ones with the highest number of articles were health and economic factors, including 37 articles and 23 articles, respectively. Psychological factors mainly included depression and mental state, and 6 articles were included. Both the family factors and the social factors constitute three subfactors each, with different elements under these subfactors. Family factors were highlighted, as the number of adult children (n = 9) and the living arrangements of older people (n = 9) were discussed more frequently. Studies outside of China identified more caregiver factors (including 3 articles), but only one Chinese study mentioned caregivers [60]. In addition, a total of four articles explored community factors that influence the need for integrated care among older people; in particular, the article by Duan and Lu [65] addressed infrastructural facilities, neighbourhood relations, and organizational structure. Social factors comprise three subcategories and were discussed in 12 articles (***Table 3***).

**Table 3:** Summary of factors influencing the integrated care needs of older adults.


CATEGORY	ACTUAL INFLUENCING FACTORS	

Demographic factors	Age [40,41,44,48,50,51,55,60,61,62,63,70,72,76,78,81,83,84,85], Gender [2,25,40,42,44,46,51,63,64,71,72], Educational attainment [2,40,41,46,48,54,60,61,63,65,67,69,77,84,85], Pre-retirement occupation [2,46,60,61,67,69,84], Marital status [2,42,60,62,63,69,71,75,85], Household registration type [76], Ethnicity [45], Residence location [60], English proficiency [42], Religious beliefs [49]	

Personal factors	Living condition	Quality of life [68], Diet quality [45], Housing quality [45], Current nursing arrangements [2], Length of stay in long-term care institutions [51]

	Personal attitudes	Health literacy [49], Purpose in Life [44], Eldercare expectations [2,46,76,78], Eldercare satisfaction level [66], Knowledge of integrated care [2,62,67,75]

	Health	IADL [40,44,45], ADL [40,41,44,48,57,80,82], ADL caregiving hours [42], Mobility difficulty [25], Level of disability [54,64], Cognitive impairment [42,56,57,64,68], Health status [2,5,40,41,45,46,53,62,63,65,66,67,70,72,76,77,81,84,85], Self-care ability [2,46,47,81,83], Number of illnesses [47,48,55,62,65,70,75,81], Disease burden [66,73], Frailty score [48], Number of medical diagnoses [71,75], Hospital admissions [48], Care dependency [59]

	Economic	Willingness to pay for integrated care [2,5], Monthly income [2,55,62,65,67,75,78,82], Financial status [41,46,60,61,63,76,77,84,85], Insurance status [2,41,42,53,66,69,70,85]

	Social intercourse	Daily leisure and entertainment [79], Number of friends seen per week [79]

Psychological factors	Depression [45,57,59], Mental state [59,77,81,84]	

Family factors	Adult children	Degree of support [5,66,73], Number [2,5,60,61,63,65,67,71,85], Distance of Residence [61], Gender [61], Family visits [58,61,65,72], Relationship [61], Degree of filial piety [61]

	Primary caregiver	Self-efficacy [42], Health [43], Burden [68], Category [60]

	Support and help	Living arrangements [46,50,62,63,64,66,70,75,77], Relationships between family members [85], Old-age expenses payer [58], Availability of same informal care in the future [43], Long wait time for help or support [43], Levels of informal assistance [54]

Community factors	Service quality [44], Infrastructural facilities [65], Neighbourhood relations [65,70], Organizational structure [65], Convenience of access to clinics [67]	

Social factors	Institutions and services	Number of types of long-term care services [5], Number of physicians per 1,000 county residents [41], Medical and nursing skill level [69], Use of medical management by a physician [50], Types of integrated care institutions [47,58], Number of skilled nursing facilities [41], Location of integrated care institutions [58], Characteristics of integrated care institutions [47,52]

	Social Welfare	Social support [40], Level of health care empowerment [64], Social welfare assistance [71], Levels of assistance received [53]

	Region/Economy	Urban GDP [78], County-level enabling [41,69]


## Discussion

Our scoping review included 49 studies covering the integrated care needs of older people aged 60 and older in 11 countries. We noted a considerable degree of similarity in older adults’ needs and factors influencing across the countries, although there were a few clear differences. The integrated care needs of the older adults in each country comprise eight categories: basic life, medical and nursing, rehabilitation, ancillary, psycho-spiritual support, social participation, health education, and welfare and aid. The factors affecting these needs fall into six categories: demographic, personal, psychological, family, community and social. This review adds an important new perspective to the broader body of knowledge on coordinated person-centred integrated care research [19]. In addition, our summary and classification of factors influencing integrated care was based on an adapted and extended WHO framework of determinants of active ageing [24], which can also inform future research.

Our review found that many studies outside of China were more multifaceted and holistic in their investigations than those in China. First, in terms of research subjects, several international studies have studied the perspective of older people and their caregivers [41,43,59,68,80], adopting a multifaceted and multiangle approach to understanding the needs of older people. In contrast, Chinese studies examined the perspective of older people only, resulting in a one-dimensional research focus. The perspective of caregivers was largely ignored. However, this perspective is important because caregivers are instrumental in connecting older adults to supportive services and negotiating services on their behalf [54]. It is therefore important to conduct a multifaceted assessment that accounts for the perspectives of not only older adults and their carers but also health care providers [33] to make the assessment of the integrated care needs of older adults more comprehensive. Furthermore, future surveys on caregivers of older adults should be differentiated according to different caregiving scenarios and the type of carer, as the responses of informal caregivers may differ from those of formal caregivers. Second, the integrated care needs assessment tools used in Chinese studies are nearly exclusively self-compiled questionnaires that are not validated or widely disseminated, and the physical and psychological status of older participants are rarely assessed using professional scales, with only five studies assessing the ability to perform activities of daily living and the health status of older people using well-established scales [55,62,73,74,78]. International scholars not only tend to use well-established scales such as The Camberwell Assessment of Need for the Elderly [48,57,59,63,80] but also employ the Katz Index of Independence in Activities of Daily Living [40,48] or the Activities of Daily Living section of the Older Americans Resources and Services Multidimensional Functional Assessment Questionnaire (ADL-OARS) [42] to assess older adults’ ability to perform activities for daily living and the Mini-Mental State Examination (MMSE) [53,57,59,80] to measure their mental state, please see details in Appendix 1. Therefore, Chinese scholars should use more well-established scales or multiple scales to assess the integrated care needs of older people to ensure the objectivity and scientific validity of the assessment.

We also found that foreign studies began to differentiate between met and unmet needs [25,48,52,57,59,80] at an early stage. Allin et al. [86] organized subjective unmet needs into five categories: (a) unperceived unmet; (b) subjective, chosen unmet; (c) subjective, not-chosen unmet; (d) subjective, clinician-validated unmet, and (e) subjective, unmet [42]. However, none of the integrated care needs assessments in the included Chinese studies were refined or differentiated. The extent of unmet needs or the extent to which require assistance is unavailable or insufficient is an important issue in public policy and the financing of health and support services [87]. The met and unmet integrated care needs of older people should be differentiated, and a distinction should even be made between utilization, potential future use, and rejection [43]. Phased implementation of a preintegration needs survey and a feedback-based needs survey after integrated care services are implemented should be performed.

At present, empirical research on the need for and willingness to accept integrated care in China is neither up-to-date nor sufficient [5]. The existing needs assessment results are similar and not sufficiently innovative, and the boundaries of the needs dimensions are also vague. There is no scientific, detailed tool for assessing integrated care needs at the national level. Our review also found that integrated care needs assessments have not yet been conducted regularly in Poland, Germany [57]. To better provide services, each state in the US regularly applies systematic methods to assess and evaluate the community service requirements of older adults [88]. Asking service users to define their needs and preferences as a means to shape policy and practice has gained credibility in several studies [52]. Quantifying the intention to use integrated care, such as LTC facilities, is useful for planning integrated care services. Moreover, when developing such services, it is critical to forecast future demand, and intention should be considered an important factor influencing the actual use of services [89]. Integrated care needs change over time; unfortunately, little longitudinal data are available to capture these changes systematically. Therefore, all countries should conduct regular needs assessments of older people within or outside of integrated services and adopt appropriate measures according to their changing needs.

In addition, as China’s integrated care needs assessment of the older population is not yet systematic, resulting in crossover and overlap among medical, nursing, health care and rehabilitation services, many scholars are accustomed to randomly combining these four types of services to conduct research, and there is no unified definition of integrated services [55]. In China, there are only pilot guidelines for the management of integrated care institutions [39], and there is a lack of standards for defining and regulating service content for community or medical institutions. In addition, boundaries between the integrated care needs of disabled older adults and the general older population are often blurred, as many in the general older population also suffer from multiple chronic diseases [74]. The concept of integrated care in the context of the perennial separation of health care services in China still faces a number of challenges. The practices required by current policies are fragmented and undeveloped, and a detailed, operational and universal set of practices has yet to be proposed. It is therefore imperative that national and local authorities not only consider the desires and needs of older adults before formulating and implementing plans for integrated care but also establish laws, systems and management systems for integrated care that are suited to China’s national conditions, thereby providing older adults with services that are both suitable and guaranteed.

In general, basic life care and medical-nursing care are the main focuses of integrated care for older adults. In the category of basic life needs, self-care, housekeeping and meal preparation were the services that emerged most often in the studies included in the review. The main reason for this result is that the deterioration of older adults’ physical functions and decrease in energy make it increasingly difficult for older adults to perform household chores [25]. When family members are working or otherwise unable to stay at home with an older adult relatives, many older adults who lack daytime surveillance and care require temporary services such as adult day care and respite care [41,42,43,50,54]; such services meet not only the basic needs of older adults but also their social and emotional needs. In addition, some older adults must provide informal support to their partners, children, grandchildren, and others, but sometimes they are powerless; in these cases, it is necessary to help them or provide replacement support services [48,59,63]. In terms of medical and nursing needs, merely improving the traditional hospital-centred care model will not adequately meet the unique healthcare needs of homebound older adults. Most older adults still want doctors and nurses to provide care services at home, such as home health care. A new diagnosis typically makes older adults aware of their physical condition and leads them to change their health care behaviour and daily life habits. Therefore, basic medical services such as disease diagnosis and treatment, regular physical examinations, and preventive health care are highly necessary for this population. Moreover, declines in memory and vision cause older adults to forget to take their medicine or to fail to understand how to take it correctly. Services that provide older adults correct, on-time medication distribution and supervision are also welcomed by this group [56]. Despite the decline in their physical function and memory, older adults do not want to lose the right to be autonomous and informed [59]. Medical information must be provided to older patients and explained clearly. Compared with older adults in other countries who require adaptive equipment [40,50,52,54], older adults in China are more willing to maintain their health by taking traditional Chinese medicine [55,73]. Furthermore, rehabilitation is very important for the recovery of damaged systems in older adults. It is necessary not only to provide services such as sports therapy, occupational therapy, acupuncture, cupping, and massage but also to provide scientific and correct rehabilitation guidance to older people and their families to maintain the continuity and effectiveness of rehabilitation treatment and health care. Surprisingly, however, many Chinese scholars have found that older Chinese people are also increasingly receiving “imported services” such as palliative care [49,76,78,79]. Finally, in terms of ancillary needs, seniors outside of China tend to prefer transportation [25,40,42,44,45,52,54,70] and money management services [25,52,59,63,70] more than Chinese seniors do. Regardless of nationality, older people require shopping, older adults hotline and on-site services [43,55]. Clearly, these three services can improve their quality of life.

With improved material and economic conditions, the number of people requiring spiritual comfort and psychological counselling has increased [58]. The older people in this study had psychosocial needs, including psychological and behavioural needs, social support needs and the need for support from informal carers, all of which are priorities in an integrated approach to care [4]. Because “home” provides emotional sustenance, older adults who live in care institutions want organizations to be considerate of them so that they can feel the warmth of home in an institutional care setting [51,81]. After retired persons leave their work unit and return to the family, their life will gradually become monotonous and boring; they will lose some social support and have difficulty maintaining close relationships. Therefore, older adults are highly interested in cultural and entertainment services (CES) and exercise [62,78]. These activities not only benefit older adults’ physical and mental health but can also promote older adults’ social participation and interpersonal communication to a certain extent, creating a virtuous circle. Furthermore, older adults want to be more educated about their disease and how to manage adverse outcomes, and overall, both older adults and their family caregivers perceived a need for more education and training on health literacy and medications [68]. Preventing misinformation among older patients is achieved through a variety of educational means and allows them to avoid being deceived in matters concerning their medical treatment under certain circumstances. Welfare and aid are mainly embodied in social welfare programmes such as medical insurance, endowment insurance, government subsidies and various aspects of legal support provided for older adults. Welfare and aid needs are the same for older people in China and worldwide. The government and society should understand the real needs of older adults in advance and tailor services to the needs of each individual, with basic living care and health education activities for those who can take care of themselves, supplemented by basic medical and rehabilitation services. Additionally, promoting cultural and educational activities and enhancing social welfare and assistance are also important. As the needs of older people are multifaceted, integrated care for older people with complex needs requires the collaboration of multidisciplinary teams or departments.

The factors influencing the need for integrated care in older people are broad and complex, involving several different aspects. Demographic characteristics and personal factors are the main factors influencing the integrated care needs of older people, with family and social factors ranking second, suggesting to some extent that the proportion of older people relying on their children to provide family care has declined. Due to globalization and the pursuit of self-fulfilment, geographical distance and life and work pressures make it difficult for young people to provide financial, emotional and caregiving support for their parents; hence, older Chinese people have changed their caregiving expectations, and the value of traditional filial piety that required absolute obedience from children has changed [90]. Age, gender, and education are the most frequently cited determinants of the demographic factors that influence the need for integrated care among older people. In the context of long-term care, age can also be rated as a further need characteristic, as it normally increases difficulties in daily living [43], which was evident in many studies. Notably, in our review, more studies conducted abroad than in China that found significant gender differences in the integrated care needs of older people. Although the reasons for this finding are unclear, in general, the needs of male and female older people in different integrated care services or in different care scenarios show significant differences; for example, male older people in the community are more eager to utilize more recreational services than female older people [71], and male older people in integrated care facilities have lower levels of all service needs than female older people [72]. In terms of personal factors, health is the most important factor that affects older people’s choice of integrated care, followed by economic situation. The economic dimension has become a main concern among older people because it is related to their willingness to pay for integrated care [2,5] and the extra financial burden of maintaining a healthy lifestyle [33]. Certainly, the living conditions, personal attitudes and social intercourse of older adults cannot be ignored. The tendency toward depression and the mental state of older adults living in long-term care facilities have also been shown to impact the need for integrated care, and the actual needs of this population are often difficult for formal caregivers such as nursing staff in care facilities to understand and meet [59].

Among family factors, older people with fewer children or who live alone show a greater need for integrated care [2,62], and those with only one child have a much higher need for health care than those with two or more children [5,85]. Regarding community factors, in addition to the relationship between older adults and their neighbours [65,70], community infrastructure and service quality mainly affect the integrated care needs of older adults [44,65]. The study by Ewen et al. [44] clearly indicates that higher demand for the HCBS was associated with older people living in service-poor housing. Social factors are complex, as the provision of services by hospitals and nursing homes, the provision of social security and welfare by the government, and even the economic level of a region affect the integrated service needs of older adults. Moreover, with the introduction and promotion of China’s policy on integrated care services, many older adults have gradually become aware of and interested in integrated services, and older people have gradually started to consider social factors such as the medical and nursing skill level [69], the type of integrated care institution [47,58] and the area where integrated care institutions are located [58].

Regardless of country or region, as the community and family are the most basic spheres of human life, people prefer to age at home or in the community rather than in an institution [2,45,91]. Our review confirmed that the community-home care scenario best meets the needs and expectations of older people. There is a wealth of research outside of China on the needs of older people in the community [91] and a preference for defining integrated care as an initiative by older people seeking to structure and coordinate care in the home environment according to their own needs [92]. Furthermore, many states in the US are attempting to redistribute Medicaid-based care from nursing homes to community settings [93], with an increasing number of integrated care programmes for older people living at home [92] and an increasing number of people living in care settings other than nursing homes. According to a WHO report (2009), an appropriate balance between care settings for older persons, including supported self-care and home-based services, is necessary. The report emphasizes the need for specific interventions to help keep older people at home and to prevent long-term institutional care [94]. Therefore, the integrated community-based home care model is likely to become a mainstream model of ageing, both in terms of the expectations of older people and in terms of international success stories and trends.

Moreover, our literature search also revealed that there were few studies conducted through “LTC institutions” as care scenarios in China [47,58,72,76,81,84], with more articles still focusing on home- and community-based care. This finding indicates that although China’s current integrated care model and policies cover nursing homes and hospitals [2], the medical institution-based approach is difficult to implement and does not address current needs, and older Chinese people, like their foreign counterparts, are more eager to age in place. However, in China, there is a clear imbalance in the distribution of existing older adult care services among the home, communities and institutions, with various preferential policies and incentives introduced by local governments favouring institutional care for older adults. Indeed, institutional older adult care services are growing rapidly and beginning to dominate, with very limited and slow development of home and community-based services [95]. Community-based home care is more reflective of the traditional Chinese family model of ageing, and its relatively low cost should become the backbone of the entire older adult care system, with older adult care institutions and medical institutions becoming complementary. Therefore, policy support and financial subsidies should be directed more towards the development of home and community-based older adult care while providing older adult care services that truly meet the physical and mental needs of older users and facilitate ageing-in-place [44], thus helping to reduce the vacancy rate of beds in older adult care institutions, the waste of older adult care and medical resources, and the problem of bed shortages in hospitals.

A strength of this study is the rigorous and systematic process of the literature search, which was conducted following Arksey and O’Malley’s guidelines [32]. To our knowledge, these findings provide a unique overview of all reviews conducted to date that outline the integrated care needs of older adults and the influencing factors. In addition, the conclusions we reached resonated with the stakeholder groups, which helped to validate the results. Despite a carefully devised research question and design, several challenges were encountered. First, as our scoping review required both demand and impact factors to be included in the literature reports, none of the qualitative studies we found met the established inclusion criteria and were thus excluded, so the studies in our review were nearly entirely quantitative. Quantitative studies sometimes raise problems, such as overemphasis on sample representativeness and lack of depth, inability to measure complex and dynamic humanities and social sciences phenomena, or failure to identify new theories [96]. The inability to derive more hidden information and needs from specific interviews with older adults is a shortcoming of our study. Second, due to differences in national circumstances, economies and ageing processes, there are differences in the integrated care, health care and older adult care systems at home and abroad, which may lead to differences in the integrated care needs of older people. Moreover, a few articles provide neither brief nor detailed terminological explanations for each need listed, so our own subjective ideas (e.g., primary and secondary classifications of integrated care needs and influences) inevitably contributed to the summary of integrated care needs and influences. However, our categorization of integrated care needs and their influencing factors were discussed and agreed upon after detailed reading of each article and by all research team members. As this study focused on summarizing the integrated needs of older people and their influencing factors, both nationally and internationally, and identifying similarities and differences, this issue does not significantly affect the findings or objectivity of this review.

## Conclusion

The review fulfilled the original objectives, answered the questions posed, and identified and summarized the specific needs of older adults in China and abroad for integrated care and the factors influencing them. There are both similarities and differences in the integrated care needs of older people at home and abroad. We also found a large gap between China and the international community in terms of integrated care and even the overall demand for older adult care services. The current research methods and perspectives on the demand for integrated care among older adults in China are still relatively homogeneous, inadequate and unstructured. The results of this review will not only serve as a reference for domestic and international researchers and policy makers but also clarify the direction of efforts and improvements. In brief, there is a long way to go in providing suitable integrated care for older adults in China.

## Additional File

The additional file for this article can be found as follows:

10.5334/ijic.5946.s1 Appendix 1. Characteristics of the included studies.
