# Supplementary material for: Older Adults’ Demand for Integrated Care and Its Influencing Factors: A Scoping Review
Source: Int J Integr Care. 2021 Dec 6;21(4):28. doi: 10.5334/ijic.5946 (PMC8663746; doi:10.5334/ijic.5946)
Supplement: Appendix 1. — Characteristics of the included studies. [file ijic-21-4-5946-s1.pdf]

Appendix

Appendix 1. Characteristics of the included studies

First Author and year	Country	Study design	Data collection methods	Integrated care needs assessment tool for older people	Study population and sample size	Care scenario
AbiHabib (2011) [63]#	Lebanon	Cross-sectional study	Survey	1. The Camberwell Assessment of Need for the Elderly<br>2. The EuroQol Five Dimensions Questionnaire (EQ-5D)<br>3. A sociodemographic tool	Older adults, 322	Home and Community
Alkema (2006) [40]#	US	Cross-sectional study	Telephone follow-up	1. The Katz Index of Independence in Activities of Daily Living<br>2. Lawton's Instrumental Activities of Daily Living Scale<br>3. Self-reported medical diagnoses or conditions	Older adults, 224	Home and Community
Beeber (2008) [41]#	US	Cross-sectional study	Data from the National Longitudinal Caregiver Study (NLCS)	1. The Older Americans' Resources and Services (OARS) Multidimensional Functional Assessment Questionnaire<br>2. The Behavior Rating Scale-Dementia (BRS-D)<br>3. The Duke OARS Comorbidity Questionnaire	Older adults and informal caregivers, 1813	Home and Community
Casado (2012) [42]#	US	Cross-sectional study	Telephone follow-up	1. The Activities of Daily Living section of the OARS Multidimensional Functional Assessment Questionnaire	Informal caregivers. 146	Home and Community

				(ADL-OARS)<br>2. The Caregiver Burden subscale of the Revised Caregiving Appraisal Scale (CB-RCAS)<br>3. The Informant Questionnaire for Cognitive Decline in the Elderly (IQCODE)<br>4. The Abbreviated Lubben Social Network Scale (A-LSNS)		
Dorin (2014) [43][#]	Germany	Cross-sectional study	None	Self-compiled questionnaire	Older adults and informal caregivers, 1152	Home and Community
Dubuc (2011) [64][#]	Canada	Longitudinal study	Data from the PRISMA study	The Functional Autonomy Measurement System (SMAF)	Older adults, 746	Home and Community
Duan (2019) [65][#]	China	Cross-sectional study	Survey	Self-compiled questionnaire	Older adults, 253	None
Ewen (2017) [44][#]	US	Cross-sectional study	Telephone follow-up, survey	1. Scales assessing psychosocial well-being<br>2. The Medical Outcomes Survey (MOS)<br>3. Provisions of social relationships subscales<br>4. The Positive and Negative Affect Scales<br>5. The Ryff Scales of Psychosocial Well-Being<br>6. The Piedmont Health Survey<br>7. The Loyola Generativity Scale	Older adults, 663	Home and Community

Ferris (2016) [45]#	US	Cross-sectional study	Data from the 2008 Southeastern Pennsylvania Household Health Survey conducted by the Philadelphia Health Management Corporation (PHMC)	1. Philadelphia Health Management Corporation (PHMC) data<br>2. The 10-item Centers for Epidemiologic Studies Depression Scale	Older adults, 1343	Home and Community
Feng (2019) [66]#	China	Cross-sectional study	Survey	Self-compiled questionnaire	Older adults, 610	Home and Community
Fan (2019) [67]*	China	Cross-sectional study	Survey	Self-compiled questionnaire	Older adults, 400	None
Gu (2020) [46]#	China	Cross-sectional study	Survey	Self-compiled questionnaire	Older adults, 408	Home and Community

Harrison (2014) [68]#	Australia	Cross-sectional study	Survey	1. Self-compiled questionnaire, 76 items from the Care Needs Assessment Package (Version-2)<br>2. A 22-item questionnaire devised for carers of stroke patients<br>3. Number and hours of current formal services<br>4. The Global Deterioration Scale<br>5. The participant and carer versions of the 13-item QOL-AD<br>6. The Neuropsychiatric Inventory<br>7. The twelve-item version of the self-report General Health Questionnaire<br>8. The Zarit Burden Interview<br>9. The Short-Form Health Survey	55 older adults, 37 informal caregivers	Home and Community
Han (2018) [47]*	China	Cross-sectional study	Survey	Self-compiled questionnaire	Older adults, 350	Long-term care institutions
Hoogendijk (2014) [48]#	Netherlands	Cross-sectional study	Survey	1. PRISMA-7<br>2. The Camberwell Assessment of Need for the Elderly<br>3. The Katz-15 Index of Independence in ADL	Older adults, 1137	Home and Community
Huang (2018) [49]*	China	Cross-sectional study	Survey	Self-compiled questionnaire	Older adults, 480	None
Huang (2017) [69]#	China	Cross-sectional study	Survey	Self-compiled questionnaire	Older adults, 511	None

Kato (2009) [50][#]	Japan	Cross-sectional study	Data from the Japan Tokyo LTCI system in April 2005	1. A questionnaire assessing current physical and mental status (73 items)<br>2. Medical procedures (12 items)	Older adults, 624	Home and Community
Kelly (2019) [51][#]	Ireland	Cross-sectional study	Survey	The Person-centered Climate Questionnaire-Patient (PCQ-P)	Older adults, 56	Long-term care institutions
Leutz (2007) [52][#]	US	Mixed methods study	None	Self-compiled questionnaire	Older adults, 800	Home and Community
Lehning (2013) [70][#]	US	Cross-sectional study	Data from the Detroit City-Wide Needs Assessment of Older Adults	Self-compiled questionnaire	Older adults, 1099	Home and Community
Li (2006) [53][#]	US	Cross-sectional study	Data extracted from the 1999 National Long-Term Care Survey (NLTC) conducted by the Center for Demographic Studies at Duke University	1. The Mini-Mental State Examination (MMSE)<br>2. A self-compiled questionnaire	Older adults, 275	Home and Community

Li (2004) [54][#]	US	Cross-sectional study	Data from Family Caregiving in the US, a national survey conducted by the National Alliance for Caregivers and the American Association of Retired Persons	None	Informal caregivers, 157	Home and Community
Li (2011) [71][#]	China	Cross-sectional study	Data from the records of nursing staff in community-based LTC facilities	1. Self-compiled questionnaire based on Andersen's model of healthy behaviour<br>2. The Barthel Index	Older adults, 200	Community-based LTC facilities
Li (2019) [72][#]	China	Cross-sectional study	Survey	Self-compiled questionnaire	Older adults, 211	Long-term care institutions
Liu (2017) [55][*]	China	Cross-sectional study	Survey	1. Self-compiled questionnaire<br>2. Chinese version of the Concise Health Questionnaire (SF-36) from the Boston Health Institute, US<br>3. Common assessment scales for ADL<br>4. The Barthel Index	Older adults, 210	Home and Community
Liu (2019) [73][#]	China	Cross-sectional study	Survey	1. The Barthel Index<br>2. Self-compiled questionnaire	Older adults, 981	Home and Community

Ling (2020) [74][#]	China	Cross-sectional study	Survey	1. The need for community support service for the disabled elderly questionnaire<br>2. The Barthel Index	Older adults, 298	Home and Community
Li (2016) [5][#]	China	Cross-sectional study	Survey	Self-compiled questionnaire	older adults, 400	None
Qiu (2020) [75][#]	China	Cross-sectional study	Survey	Community Medical-Nursing Combined Service (MNCS) Demand Questionnaire	Older adults, 120	Home and Community
Sandberg (2019) [56][#]	Sweden	Cross-sectional study	Data from one agency in Sweden	Self-compiled questionnaire	Older adults, 131	Home and Community
Si (2020) [76][#]	China	Cross-sectional study	Survey	Self-compiled questionnaire	Older adults, 1907	Long-term care institutions
Tobis (2018) [57][#]	Germany	Cross-sectional study	Survey	1. The Mini-Mental State Examination (MMSE)<br>2. The Barthel Index<br>3. The Geriatric Depression Scale (GDS)<br>4. The Camberwell Assessment of Need for the Elderly	Older adults, 306	Long-term care institutions
Tian (2017) [77][*]	China	Cross-sectional study	Survey	Self-compiled questionnaire	Older adults, 300	Home and Community
Tian (2020) [58][#]	China	Cross-sectional study	Survey	Self-compiled questionnaire	Older adults, 331	Long-term care institutions

van den Brinkrink (2018) [59]#	Netherlands	Longitudinal study	Chart review, semi-structured interviews, (brief) neuropsychological testing, and survey	1. The Camberwell Assessment of Need for the Elderly<br>2. The Mini Mental State Examination (MMSE)<br>3. The Revised Index for Social Engagement (RISE)<br>4. The Care Dependency Scale (CDS)<br>5. The Geriatric Depression Scale (GDS-8)<br>6. The Hospital Anxiety and Depression Scale (HADS-A)<br>7. The Dutch informant personality questionnaire (the HAP)	Older adults and formal caregivers, 141	Long-term care institutions
Wang (2020) [2]#	China	Cross-sectional study	Survey	Self-compiled questionnaire	Older adults, 7320	None
Wang (2018) [78]#	China	Cross-sectional study	Survey	1. The Barthel Index<br>2. Self-compiled questionnaire	Older adults, 298	Home and Community
Wang (2020) [79]#	China	Cross-sectional study	Survey	Self-compiled questionnaire	Older adults, 374	None
Wei (2020) [60]#	China	Cross-sectional study	Survey	Self-compiled questionnaire based on Andersen’s model of healthy behaviour	Older adults, 3260	Long-term care institutions

Wieczorowska-Tobis (2016) [80]#	Germany	Cross-sectional study	Survey	1. The Camberwell Assessment of Need for the Elderly<br>2. The Barthel Index<br>3.The Mini-Mental State Examination (MMSE)<br>4. The Geriatric Depression Scale	Older adults and formal caregivers, 173	Long-term care institutions
Wilkinson-Meyers (2014) [25]#	New Zealand	Cross-sectional study	Telephone follow-up, survey	1. The World Health Organization's Quality of Life measure (WHOQOL-BREF)<br>2. The Nottingham Everyday Activities of Daily Living (NEADL)	Older adults, 3753	Home and Community
Wu (2020) [81]#	China	Cross-sectional study	Survey	Self-compiled questionnaire	Older adults, 300	Long-term care institutions
Xu (2018) [61]#	China	Cross-sectional study	Survey	Self-compiled questionnaire	Older adults, 288	None
Xu (2018) [82]#	China	Cross-sectional study	Survey	Self-compiled questionnaire	Older adults, 210	Home and Community
Yuan (2020) [62]*	China	Mixed method study	Survey	1. The Barthel Index<br>2. Self-compiled questionnaire	Older adults, 460	Home and Community
Zhao (2020) [83]#	China	Cross-sectional study	Survey	Self-compiled questionnaire	Older adults, 175	Home and Community
Zhu (2016) [84]#	China	Cross-sectional study	Survey	Self-compiled questionnaire	Older adults, 106	Long-term care institutions
Zhang (2017) [85]#	China	Cross-sectional study	Survey	Self-compiled questionnaire	Older adults, 366	None

Note. #= Journal article, *= Dissertation; An informal caregiver is a family member or friend. A formal caregiver is a qualified nurse or nursing staff.
